# Bisphenols as a Legacy Pollutant, and Their Effects on Organ Vulnerability

**DOI:** 10.3390/ijerph17010112

**Published:** 2019-12-22

**Authors:** Jong-Joo Kim, Surendra Kumar, Vinay Kumar, Yun-Mi Lee, You-Sam Kim, Vijay Kumar

**Affiliations:** 1 Department of Biotechnology, Yeungnam University, Gyeongsan, Gyeongbuk 38541, Korea; kimjj@ynu.ac.kr (J.-J.K.); ymlee@yu.ac.kr (Y.-M.L.); samsam5332@naver.com (Y.-S.K.); 2 Department of Anatomy, All India Institute of Medical Sciences, New Delhi 110029, India; surendrakhedarcbt@gmail.com; 3 Department for Management of Science and Technology Development, Ton Duc Thang University, Ho Chi Minh 758307, Vietnam; vinay.kumar@tdtu.edu.vn

**Keywords:** bisphenols, endocrine disruptors, obesity and diabetes mellitus, hepatic toxicity, neurotoxicity, immunotoxicity

## Abstract

Bisphenols are widely used in the synthesis of polycarbonate plastics, epoxy resins, and thermal paper, which are used in manufacturing items of daily use. Packaged foods and drinks are the main sources of exposure to bisphenols. These chemicals affect humans and animals by disrupting the estrogen, androgen, progesterone, thyroid, and aryl hydrocarbon receptor functions. Bisphenols exert numerous harmful effects because of their interaction with receptors, reactive oxygen species (ROS) formation, lipid peroxidation, mitochondrial dysfunction, and cell signal alterations. Both cohort and case-control studies have determined an association between bisphenol exposure and increased risk of cardiovascular diseases, neurological disorders, reproductive abnormalities, obesity, and diabetes. Prenatal exposure to bisphenols results in developmental disorders in animals. These chemicals also affect the immune cells and play a significant role in initiating the inflammatory response. Exposure to bisphenols exhibit age, gender, and dose-dependent effects. Even at low concentrations, bisphenols exert toxicity, and hence deserve a critical assessment of their uses. Since bisphenols have a global influence on human health, the need to discover the underlying pathways involved in all disease conditions is essential. Furthermore, it is important to promote the use of alternatives for bisphenols, thereby restricting their uses.

## 1. Introduction

Bisphenols (Bisphenol A (BPA), Bisphenol B (BPB), Bisphenol C (BPC), Bisphenol S (BPS), Bisphenol F (BPF) and Bisphenol AF (BPAF)) are phenolic organic compounds (Table 1). These compounds are commonly used in the manufacturing of plastic containers, epoxy resins, food and drink cans, water pipes, electronic equipment, thermal paper, kitchen utensils, toys, and dental sealants [1,2,3,4,5]. Bisphenols are generally used for the hardening of plastic and are easily dissolved in foods and drinks. The general population is therefore exposed to bisphenols, both directly (through oral and/or topical routes) and indirectly (through environmental pollution and/or food chain) [5,6,7,8,9,10]. Due to their extensive uses and long-term discharge from plastic products, humans always have a concentration of bisphenols in their body fluids, even without intentional exposure [11,12].

Bisphenols exert toxic effects due to their potential to induce oxidative stress, mitochondrial dysfunction, impaired inflammatory function, and endocrine disruption activity. Bisphenol exposure leads to mitochondrial dysfunction, deregulation of cellular signaling pathways, and generation of reactive oxygen species (ROS) subsequent to decreased antioxidant enzymes [5,13,14,15,16,17,18,19,20]. Elevated ROS levels result in oxidative stress, DNA damage and cell death, by activating the caspase cascade as well as mitogen-activated kinases (MAPK) signaling pathways [16,19,20,21,22,23,24,25]. Bisphenols induce inflammatory responses via various signaling pathways and alteration of various immune cells. Bisphenol exposure stimulates the production of pro-inflammatory cytokines and inhibits the production of anti-inflammatory cytokines [20,23,26]. Human studies also have determined that bisphenol toxicities are associated with oxidative stress and inflammatory responses [27,28,29,30].

Bisphenols bind to androgen, estrogen, progesterone, thyroid, and aryl hydrocarbon receptors, which are, in turn, associated with endocrine and other systems of the body, especially the reproductive, respiratory, and nervous systems [31,32,33,34,35]. Exposure to bisphenols disrupts the activity of several hormones, including sex hormones, insulin, and thyroxin, causing different organ toxicities [15,36,37]. Hence human exposure to bisphenols has increased the risk of obesity, diabetes mellitus, liver dysfunction, cardiovascular diseases, reproductive, and developmental abnormalities [38,39,40,41]. Bisphenols are metabolic disruptors, and even early-life exposure at low concentrations can result in impaired metabolic functions and toxicity to several organs or systems. These complications are further reviewed in the following subsection. Moreover, BPA is known to interact with therapeutic drugs and may affect the outcomes of chemotherapy [42].

## 2. Endocrine Disruption and Reproductive Abnormalities

Bisphenol toxicity studies have revealed that these chemicals disrupt the endocrine functions and cause reproductive toxicity [43,44,45,46]. Even a low dose of chronic exposure to bisphenols suppresses the luteinizing hormone, follicle-stimulating hormones, and prolactin, exhibiting estrogenic and antiandrogenic effects and affecting spermatogenesis [12,47,48]. Common reproductive abnormalities include premature puberty, ovarian dysfunction, implantation failure, abnormal sperm function, fertilization failure, sex hormone abnormality, premature birth, and lower birth weight [48,49,50,51,52]. Bisphenols interact with both membrane-bound and nuclear estrogen receptors. BPA mimics the action of the natural estrogen 17-β estradiol, and their metabolites have greater estrogenic activities [53,54,55].

In a prospective study of 1841 pregnant women, Zhang et al. found that BPAF and BPS are potential risk factors for gestational diabetes mellitus (GDM). The authors observed the endocrine-disrupting effects of BPAF and BPS on blood glucose metabolism among Chinese pregnant women. Moreover, multivariable logistic regression analysis revealed an association of urinary BPAF with the risk of GDM [56]. In a cross-sectional study on 167 men, Meeker et al. found that urinary BPA level is inversely related to the estradiol:testosterone ratio, indicating that BPA probably modulates the estrogen and androgen synthesis gene expression [37]. A positive correlation between urinary BPA level and higher expression of two estrogen-responsive genes (encoding ERβ and ERRα) were found in the European population [57]. Increased urinary BPA level has also been associated with decreased thyroid-stimulating hormone and increased levels of free triiodothyronine hormone in a Chinese population aged 40 years or older [58]. BPA, BPF, and BPS have also been detected in human breast milk samples from Spanish mothers [59,60] (Table 2).

Numerous studies in animal models have reported the deleterious effects of exposure to bisphenols. Tian et al. found that male CD-1 mice exposed to BPA had decreased testis weight, damage to basal lamina of seminiferous tubules and tight junctions between Sertoli cells, and decreased levels of the androgen-binding protein [61]. BPA exposure of pregnant CD-1 mice results in altering the tissue organization of ovaries and mammary glands, and alters the estrous cycle in adulthood via modulation of their morphogenesis specific genes’ expression [62]. Intraperitoneal administration of BPA (25 mg/kg BW /day; 9 days) to 8-week-old female Wistar rat decreased catalase expression, and increased lipid peroxidation and nitric acid levels in granulosa cells of the ovary. BPA exposure also decreases the estrogen and progesterone levels, and increases pro-inflammatory cytokine levels [63]. Sub-acute oral administration of BPA (10 mg/kg BW/day) in adult Wistar rats increases the serum estrogen level and prostate-specific antigen, causing vascular congestion and hyperplasia of the prostatic epithelium [64]. BPA at low doses decreases the synthesis of estradiol and inhibits the growth of antral follicles isolated from wild-type and Ahr knock-out mice, through partial involvement of the aryl hydrocarbon receptor pathway [32]. BPA exposure results in decreased sperm counts and motility, and increased ROS and lipid peroxidation in mice testes [25] (Table 3).

Antiandrogenic and estrogenic activities of BPF and BPAF are similar to BPA [65,66,67,68,69]. BPF exposure decreases the basal testosterone secretion by human fetal testes, and induces the production of 17β-estradiol [70,71]. Zebrafish larva exposed to BPF promotes the production of estrogen, which causes phenotypic feminization and alters sexual differentiation [72]. BPA, BPF, and BPS exposure to mouse embryonic stem cells (mESCs) disrupt numerous processes during mESC global and neural differentiation, thereby triggering the onset of cardiovascular/neural diseases and cancer [73]. BPA exposure also exerts differential effects on the mouse GC-1 spermatogonial cell line by altering the cell growth, global DNA methylation, histone level, and MAPK signaling pathways [20]. In a similar study, exposure of the mouse spermatocyte GC-2 cell line to BPA, BPF, and BPS altered the DNA methylation and steroidogenesis-related gene expressions [46]. Both BPA and BPS exposure results in modified activities of the ABCB1 promoter in human placental 3A cells, which affect the placental P-glycoprotein efflux transporter levels. Such changes in the levels of placental P-glycoprotein significantly affects fetal exposure to xenobiotics [74]. Gentilcore et al. reported that exposure of thyroid immortalized cell line (FRTL-5) and zebrafish embryos to BPA (10^−9^ M) affects the thyroid follicular cells through an altered expression of the thyroid-specific gene and transcriptional factors [75]. Sheng et al. found that low dose BPA (10^−9^ M) treatment of CV-1 cells derived from *Cercopithecus aethiopis* monkey kidney suppresses the thyroid hormone receptor transcription [76]. Neuregulins are a member of the epidermal growth factor family proteins involved in embryogenesis and the development of many internal organs. A study in a pig model reveals that low and high dose exposures of BPA (0.05 and 0.5 mg/kg BW/day) alters the number of neuregulin-1-positive fibers and their neurochemical properties, in both uterine muscular and mucosal layers [52]. BPA (2000 and 4000 µg/L) exposure to zebrafish embryo during the first 24 h of development has been shown to impair the migration of primordial germ cells [77].

## 3. Obesity and Diabetes

Studies on humans and animals have revealed that bisphenol toxicity is associated with obesity and impaired glucose homeostasis [5,82,83,96,97,98,99]. BPA exposure alters the glucose-stimulated insulin/C-peptide response in humans [100]. In a repeated-measures, longitudinal study from China, Wang et al. found that BPA exposure is associated with impaired glucose homeostasis in women (aged ≥ 40 years), and has a high urinary BPA level. However, no significant associations were found between glucose metabolic markers and urinary BPA in men [85]. By disrupting the endocrine activity, bisphenol exposure resulted in increased body weight. Bisphenols are also reported to bind to the α and β receptors of fatty tissues and modulate their functions. BPA exposure also disrupts thyroid signaling by affecting the metabolism of the thyroid hormone [101].

A prospective study from a Swedish population (*n* = 1016, mean age = 70 years) reported that serum BPA levels are positively associated with adiponectin and leptin, and inversely associated with the gut-hormone ghrelin. BPA is also shown to interfere with the hormonal control of hunger and satiety [102]. A cross-sectional study of 2838 participants in the USA reported that urinary BPA levels correlated with increased body weight in children and adolescents [83]. An epidemiological study conducted in a Chinese population found a dose-related association between high urinary BPA concentration and increased body weight in 9–12 year old girls [97]. Another study from China reported that increased urinary BPA levels of both girls and boys (aged 8–15 years) are related to increased body mass index [81]. In a Chinese population case-control study of 251 cases of diabetes mellitus type 2 (DM2) and 251 controls, urinary concentrations of bisphenols (BPAF and BPS) were found to be positively correlated with DM2 [78]. A meta-analysis study determined that BPA toxicity is related to an increased risk of DM2; they reported that both urine and serum BPA levels are positively associated with the risk of DM2 [80]. In a cross-sectional study on the middle-aged and elderly Chinese population, a positive association was found between generalized obesity, abdominal obesity, and insulin resistance [82]. In an epidemiological study in the USA, Shankar et al. found a positive association between increased urinary BPA concentration and diabetes mellites, which was independent of confounding diabetes risk factors [79] (Table 2). In a meta-analysis study, Kim et al. reported that BPA exposure in children is associated with the risk of obesity [103].

Bodin et al. found that long-term BPA toxicity accelerates spontaneous insulitis and diabetes mellitus type 1 (DM1) development in non-obese diabetic mice [87]. Zhao et al. showed that 1 and 10 μg/L BPS exposure to male zebrafish significantly increases the fasting blood glucose levels, decreases insulin levels, and impairs glucose homeostasis [96]. Liu et al. showed time and gender-dependent effects of maternal BPA (100 µg/kg/day) exposure on the body weight and glucose homeostasis disorder in C57BL6 mice offspring. These offspring have glucose intolerance and decreased insulin secretion [104] (Table 3).

## 4. Cardiovascular Toxicity

Several studies have established the role of bisphenol in cardiovascular diseases, including myocardial infarction, cardiomyopathy, and hypertensive heart disease [105,106,107,108,109]. A survey conducted in the United States shows the correlation between urinary BPA levels and increased prevalence of heart disease [84]. Shankar et al. found that increased serum BPA concentration is associated with the development of peripheral arterial disease in United States adults. The observed association was independent of their lifestyle (smoking and alcohol), body mass index, hypertension, cholesterol level, and diabetic status [106]. Another study confirmed that BPA exposure is associated with decreased heart rate variability and increased blood pressure in elderly subjects (≥60 years old). The risk of hypertension was also increased with increasing urinary BPA concentration in participants [110]. In a cross-sectional study (*n* = 1016 subjects; age = 70 years), Olsen et al. found positive associations between the serum concentration of BPA and LDL cholesterol levels [111] (Table 2).

Feiteiro et al. observed that BPA inhibits the L-type calcium channels in rat aorta smooth muscles, leading to the relaxation of vascular smooth muscles, which was found to be dependent on the concentration of BPA [94]. BPA escalates the worsening of DM1 by disrupting calcium homeostasis in the mouse pancreas, resulting in endoplasmic reticulum stress in pancreatic cells and promoting insulin resistance [86]. Perinatal exposure of bisphenols (BPAF, BPA, and BPF) regulate the expressions of hepatic glucose and lipid metabolism specific genes, and hence inhibit their homeostasis in adolescent female mice offspring [112]. Furthermore, they differentially influence oxidative damage and metabolic disorders in the livers of male mice offspring [113] (Table 3).

## 5. Hepatotoxicity

BPA exposure leads to hepatotoxicity by oxidative stress, mitochondrial impairment, and inflammatory pathways [15,114,115,116,117]. Nicolucci et al. found BPA in human plasma samples and observed an association between BPA exposure and liver health status [118].

Meng et al. found that perinatal exposure to bisphenols (BPA, BPF, and BPAF) differentially influences metabolic disorders and oxidative stress in the liver of male mouse offspring. BPF exposure affected the liver antioxidant defense system, whereas BPAF altered the level of β-glucose and glycogen [113]. Oral dosing of BPA to male Wistar rats decreases the antioxidant enzymes and their gene expressions, and increases the liver enzyme activity [15]. Similarly, Moon et al. found that mice exposed to a low dose of BPA might cause structural changes in the liver and mitochondrial dysfunction. Moon et al. confirmed that BPA induces lipid peroxidation, and decreases glutathione peroxidase activity and expression of pro-inflammatory cytokines such as tumor necrosis factor-α and interleukin-6 [13]. BPA exposure in mice also induces lipid accumulation in the hepatic cells by affecting fatty acid synthesis and their transport genes. BPA exposure leads to excessive lipid accumulation in the liver, decreases the levels of autophagy, and induces nonalcoholic fatty liver disease and their associated complications [114,115,116,117]. Long-term BPA exposure (0.5 μg BPA/kg/day, 10 months) on male mice induces hepatic lipid accumulation, which may be due to the epigenetic reprogramming of genes involved in lipid metabolism, such as alterations of DNA methylation patterns [119]. BPA (15 μg/L for 3 and 6 weeks) exposure to *Gobiocypris rarus* fish disturbed the expressions of acetyl-CoA carboxylase, fatty acid synthase, and carnitine palmitoyltransferase 1α, by altering the sterol regulatory element-binding protein 1 binding to their sterol regulatory elements, subsequently affecting triglyceride synthesis. BPA exposure led to a gender-specific effect on fatty acid β-oxidation in *G. rarus* fish [120].

Human HepG2 hepatoma cells exposed to low concentrations (10^−4^–10^−12^ M) of BPA showed mitochondrial dysfunction by inducing ROS generation, lipid peroxidation, mitochondrial transmembrane hyperpolarization, and release of interleukin-8 and tumor necrosis factor-α secretion [95] (Table 3).

## 6. Neurotoxicity

Exposure to bisphenols is associated with several neurological dysfunctions comprising memory and cognitive impairments, including aggression, hyperactivity, anxiety, depression, autism, and neuroinflammation. Children are more prone to bisphenol exposure, and even low concentrations (≤100 µM) are toxic to brain development in both prenatal and childhood stage [89,121,122,123,124,125]. Exposure to BPA in the first trimester of pregnancy is associated with sleep-related problems in preschool children [80]. In a longitudinal birth cohort study in California, Harley et al. observed that prenatal and early childhood BPA exposure of children results in behavioral problems, including anxiety, depression, and hyperactivity [123]. Evans et al. found that prenatal exposure to BPA may be related to increased behavioral problems in school-age boys, but not in girls [126]. Increased serum and urine BPA levels were detected in children with autism spectrum disorder (ASD) [124,127]. Metwally et al. observed that BPA exposure induces oxidative stress in ASD children, which results in mitochondrial dysfunction and behavior impairment [128]. In a similar study, the authors found that serum levels of follicle-stimulating hormone, inhibin B, and estradiol hormones were lower in the ASD group than the control group [129]. (Table 2).

Prenatal BPA exposure of Wistar rats causes changes in the hippocampal expressions of genes associated with ASD in a sex-specific manner. BPA disrupts the expression of ASD candidate genes (*Auts2*, *Foxp2*, and *Smarcc2*) more significantly in the male hippocampus than in females [130]. Subcutaneous injection of 20 μg BPA/kg BW/day in pregnant mice resulted in impaired neurotransmission. BPA increases the levels of dopamine as well as their metabolites, and decreases the levels of serotonin and their derivatives in the brain [131]. In a similar study on pregnant mice, prenatal and lactational BPA exposure (20 μg/kg BW/day), from embryonic day to 21st postnatal day, resulted in impaired murine behavior [132]. Chen et al. found that juvenile BPA exposure impairs the spatial memory only in male rats, in a dose and gender-dependent manner. Such cognitive impairment was due to changes of the excitatory plasticity, such as the downregulated spine density and glutamate receptor expression levels in the hippocampus [88]. High concentrations of BPA (>100 μM) exposure in mice hippocampal HT-22 cells induces apoptosis by increasing the calcium influx and ROS levels, followed by activating the phosphorylation of extracellular signal-regulated kinase, c-Jun N-terminal kinase, and caspase 3 [133]. BPA causes developmental toxicity through antiproliferation and pro-apoptosis in rat embryonic midbrain (MB) cells. Khadrawy et al. found the neurochemical impact of BPA in the cortex and hippocampus region of adult male albino rat brain. The authors found that BPA induces a state of oxidative stress and excitotoxicity-cum-acetylcholinesterase activity in these regions [92]. Long-term, low-level BPA exposure causes impaired learning and memory ability, increases the DNA damage in brain cells, and decreases the cell density in the hippocampus of adolescent mice. [134]. Low concentrations of BPA exposure block the cell cycle progression and increase the induced apoptosis. BPA exposure decreases the phosphorylation of c-Jun N-terminal kinase and cyclic-AMP response binding protein in MB cells, and increases the mRNA expressions of proapoptotic proteins (Bax and p53) [21]. Poimenova et al. conducted a study on 6-weeks old Wistar rat offsprings treated with BPA (orally; 40 µg/kg/day) during pregnancy and lactation. Results showed increased anxiety-like behavior and reduced exploratory behavior in a corticosterone-regulated manner [135].

BPS exposure at concentrations of 0.3 and 3.0 mg/L on zebrafish embryos showed decreased locomotor activity, increased oxidative stress, apoptosis, and altered retinal structure. Moreover, the researchers also found that 3.0 mg/L BPS exposure suppresses the expression of six neuro development-specific genes (*mbp*, *syn2a*, *α1-tubulin*, *elavl3*, *gap43*, and *gfap*) [91]. Kinch et al. showed that an acute low dose of BPS or BPA (0.0068 mM) exposure on zebrafish modified the growth of hypothalamus and caused hyperactive behavior [93]. A similar study showed that chronic BPS exposure on male zebrafish results in structural impairment of the retina and reduction of their tracking capability [136] (Table 3).

## 7. Immunotoxicity

Bisphenols affect inflammation and immune responses through several signaling pathways, and are capable of both initiating as well as inhibiting the activities of immune cells. Bisphenols modulate the immune response by affecting estrogenic receptors, aryl hydrocarbon receptors, and peroxisome proliferator-activated receptors. Exposure to bisphenols can alter the function of cytokines and chemokines, which exacerbates or results in immune-related diseases (e.g., allergy, asthma, multiple sclerosis) [137,138,139]. An epidemiological study conducted in the USA reports the association between higher urinary BPA levels with higher cytomegalo virus antibody titers in the <18-years age group, indicative of the negative impacts of BPA on immunity [36].

BPA exposure decreases neutrophilic activity and inhibits interleukin-6 formation in mice infected with non-pathogenic *Escherichia coli* [140]. Roy et al. showed that the offspring of female mice exposed to BPA were more susceptible to infection by the influenza A virus, which is associated with the modulation of their innate immunity [141]. T-lymphocytes of mice treated with BPA have increased secretion of interferon-γ and decreased secretion of interleukin-4 [142], whereas Lee et al. observed that BPA increases the levels of both interleukins 4 and 8 in mouse T-lymphocytes [143]. BPA (1 μM) exposure to mouse splenic lymphocytes inhibits the mitogenesis of these cells, particularly B lymphocytes [144]. Mice treated with BPA produce lymphocytes with higher amounts of immunoglobulin A and IgG2a. BPA exposure affects the nonspecific immune defenses [90] and modulates proliferation of B cells as well as the production of some cytokines and antibodies [145]. BPF and BPS exposure is reported to increase oxidative stress and the expressions of immunity-related genes in a concentration-dependent manner during the early developmental stages in zebrafish [146]. *Carussius auratus* exposed to BPA results in immunotoxicity, and such fish are prone to infectious diseases [147] (Table 3).

## 8. Discussion

Bisphenols are widely used as a raw material in the synthesis of polycarbonates, epoxy resins, and thermal paper. These chemicals are used in the manufacturing of numerous products including plastics, water pipes, toys, medical equipment, electronics, food cans, and numerous household applications. These chemicals are leached from the products and are ubiquitous in the environment. Foods and drinks are the most important sources of exposure. Results of both animal and human studies have revealed the toxic effects of bisphenols. Increased levels of bisphenols are found in human body fluids including in urine, serum, placental tissue, umbilical cord blood, and breast milk [60,78,84,97,124]. These chemicals affect animal and human organisms by interactions with estrogen, androgen, and aryl hydrocarbon receptors, and disrupt the function of the endocrine system, including altering the functions of sex hormones, insulin, leptin, adiponectin, or thyroxin. These chemicals exert various effects in living organisms as they are able to interact with receptors, generate ROS, lipid peroxidation, and alter cell signaling. Epidemiological studies have shown that exposure of the general population to bisphenol increases the risk of coronary heart diseases, neurological disorders, and metabolic disorders, including obesity and diabetes. Continuous exposure to bisphenols has shown detrimental effects on reproduction, development, and neural networks. These chemicals also affect the biology of immune cells, and play a significant role in the initiation or exacerbation of inflammatory conditions. Moreover, bisphenols show an age, gender, and dose-dependent effect. Even a low concentration of bisphenol is known to be detrimental; hence, critical assessment of their uses and the global influence on human health is necessary.

Humans and animals are exposed to bisphenols from the prenatal stage to the last day of survival. Bisphenols are present in products used daily, and we are always in contact with these chemicals, either indirectly or directly. Prenatal and childhood bisphenol exposure affects the developmental process and leads to neurological, reproductive, immunological, and endocrine disruption. Even low doses of bisphenols are toxic, and chronic exposure affects almost every body part. Bisphenols induce oxidative stress, inflammation, apoptosis, and impairs the metabolic process. Nowadays, almost every product contains bisphenol and we are unwillingly exposed to it. Foods and drinks stored in plastic containers get easily contaminated with bisphenols, and consumption of such foods results in gradual accumulation of bisphenols in our body. This accumulation eventually leads to toxicity of major body organs including the liver, brain, and kidney, ultimately disrupting the neurological, immunological, reproductive, and endocrine functions. 

## 9. Conclusions 

We need to have alternatives of bisphenols and try to minimize their uses because of their continuous exposure and impending toxicity. We have to work on processes/methods which can remove/filter out bisphenols from our body. Furthermore, a shift in focus towards natural plant products for packaging, storing, and other day-to-day activities is required. Soil made products can be promoted for storage of foods and drinks. The use of plastic bags needs to be restricted for shopping and extra packaging, and we need to promote the use of eco-friendly bags made of natural plant products.

Bisphenols have a wide range of toxicity profiles. However, we limited our discussion to the major organ systems. We did not discuss their role in cancer, respiratory system, renal, and developmental toxicity.

## Figures and Tables

**Table 1:** Chemical formula, IUPAC name, and chemical structure of common bisphenols.

Sl. No.	Bisphenol	Chemical Formula	IUPAC Name	Chemical Structure *
1.	Bisphenol A (BPA)	C_15_H_16_O_2_	4-[2-(4-hydroxyphenyl)propan-2-yl]phenol	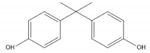
2.	Bisphenol B (BPB)	C_16_H_18_O_2_	4-[2-(4-hydroxyphenyl)butan-2-yl]phenol	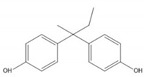
3.	Bisphenol C (BPC)	C_14_H_10_Cl_2_O_2_	4-[2,2-dichloro-1-(4-hydroxyphenyl)ethenyl]phenol	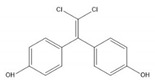
4.	Bisphenol F (BPF)	C_13_H_12_O_2_	4-[(4-hydroxyphenyl)methyl]phenol	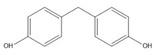
5.	Bisphenol S (BPS)	C_12_H_10_O_4_S	4-(4-hydroxyphenyl)sulfonylphenol	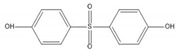
6.	Bisphenol AF (BPAF)	C_15_H_10_F_6_O_2_	4-[1,1,1,3,3,3-hexafluoro-2-(4-hydroxyphenyl)propan-2-yl]phenol	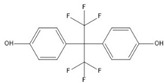

* Chemical structures were drawn using ChemDraw version 18 (PerkinElmer, MA, USA).

**Table 2:** Effect of bisphenol exposure in the human population.

Sl No.	Human Population Study	Bisphenols Studied	Finding	Reference
1	A case-controlled study (251 each)	BPAF, BPA, BPS	Urinary BPAF and BPS concentrations are positively associated with DM2	[78]
2	NHANES-2003-08	BPA	Urinary BPA levels are associated with diabetes mellitus and Peripheral Arterial Disease	[38,79]
3	485 adults (259 men, 92 premenopausal women, and 134 postmenopausal women)	BPA	Urinary BPA levels are associated with oxidative stress and inflammation in postmenopausal women	[27]
4	Case-control study	BPA, BPAF, BPS	Urinary concentrations of bisphenols are positively associated with DM2 risk	[78]
5	Meta-analysis; human population	BPA	BPA exposure is positively associated with DM2 risk	[80]
6	A cross-sectional study in Chinese school children	BPA	BPA exposure increases the BMI in school children	[81]
7	Cross-sectional study (*n* = 3390; age ≥ 40 years)	BPA	BPA is positively associated with generalized obesity, abdominal obesity, and insulin resistance	[82]
8	Cross-sectional study (*n* = 3394; age ≥ 40 years)	BPA	Urinary BPA levels are associated with increased thyroid function	[58]
9	Cross-sectional study (*n* = 2838; age 6–19 years); NHANES-2003-08	BPA	Urinary BPA levels are associated with obesity	[83]
10	Cross-sectional study (*n* = 1455 (2003–2004) and *n* = 1493 (2005–2006); age 18–74 years); NHANES-2003-06	BPA	Urinary BPA levels are associated heart disease	[84]
11	Cross-sectional study (*n* = 167 Men; age 8–55 years);	BPA	Urinary BPA levels are associated with altered serum thyroid and reproductive hormone levels in men	[37]
12	A prospective study (*n* = 1841; pregnant women)	BPA, BPS, BPF, BPAF	BPAF and BPS might be potential risk factors of gestational diabetes mellitus	[56]
13	A repeated-measures, longitudinal study (*n* = 2336, age ≥ 40); non-diabetic adults; Chinese population	BPA	BPA is independently associated with dyshomeostasis of glucose before the development of diabetes in women (age ≥ 40)	[85]

Bisphenol A (BPA); Bisphenol S (BPS); Bisphenol F (BPF); Bisphenol AF (BPAF); Bisphenol B (BPB); reactive oxygen species (ROS); mitogen-activated kinases (MAPK); diabetes mellitus type 1 (DM1); diabetes mellitus type 2 (DM2); National Health and Nutritional Examination Survey (NHANES); body mass index (BMI).

**Table 3:** Effect of Bisphenol exposure in different experimental models.

Sl No.	Study	Bisphenols	Dosing	Finding	Reference
In-Vivo Studies					
1	Streptozotocin-induced type 1 diabetes mellitus male mice model	BPA	5 mg/kg BW for 5 days, gavage	Disruption of calcium homeostasis; insulin resistance	[86]
2	Eight-week-old female Wistar rats	BPA	25 mg/kg BW/day for 9 days, intraperitoneal	Catalase plays a role in mediating reproductive damage in granulosa cells exposed to BPA	[63]
3	Non-obese diabetic mice	BPA	0, 1, and 100 mg/L BPA in drinking water	Long-term BPA exposure at a dose three times higher than the tolerable daily intake of 50 µg/kg, appears to accelerate spontaneous insulitis and diabetes development in non-obese diabetic mice	[87]
4	Male and female Sprague-Dawley rats	BPA	0.04, 0.4, and 4 mg/kg/day	Juvenile BPA exposure disturbs the spatial memory in male rats, but not in female rats, in a dose-dependent manner; alteration of the excitatory plasticity; downregulates the spine density and glutamate receptor expression levels in the hippocampus	[88]
5	Male Sprague-Dawley adult rats	BPA	40 μg/kg; subcutaneous injection	Acute BPA exposure impaired memory and block synaptic plasticity processes	[89]
6	Zebrafish (*Danio rerio*)	BPA	10^−9^ M	Alters the expressions of genes involved in thyroid hormone synthesis and of thyroid-specific transcriptional factors, in a dose- and time-dependent manner	[75]
7	T-cell receptor transgenic mice	BPA	10 mg/L, for 2 weeks; drinking water	Reduces interleukin-2, 4, and interferon γ secretions, and increases the productions of IgA and IgG2a	[90]
8	Zebrafish larvae	BPS	0, 0.03, 0.3 and 3.0 mg/L until 6 days post-fertilization	Developmental neurotoxicity; decreases locomotor behavior; promotes apoptosis, and alters retinal structure; downregulates the expression levels of neurodevelopment genes	[91]
9	Male Wistar albino rats	BPA	0.1, 1, 10, 50 mg/kg/day; oral	ROS generation; reduces antioxidant gene expression; hepatotoxicity	[15]
10	Adult male Wistar albino rats	BPA	10 mg/kg for 6 and 10 weeks, and 25 mg/kg for 6 weeks	Induces a state of excitotoxicity and oxidative stress	[92]
11	Zebrafish	BPA, BPS	0.0068 μM	Affects neurodevelopment; induces precocious hypothalamic neurogenesis	[93]
12	Zebrafish larvae	BPF	1, 10, 100, and 1000 μg/L BPF, from fertilization to 60 days post-fertilization	Developmental exposure to BPF adversely affects sexual differentiation	[72]
In-Vitro Studies					
1	Mouse embryonic stem cells (mESCs)	BPA, BPF, and BPS		Causes developmental toxicity, and may trigger the onset of cardiovascular/neural diseases and cancer	[73]
2	Vascular smooth muscle cell line obtained from embryonic rat aorta	BPA		BPA inhibits the L-type calcium channels, leading to the relaxation of aorta smooth muscle	[94]
3	HepG2 cells	BPA	10^−4^–10^−12^ M	Mitochondrial dysfunction; lipid accumulation in hepatic cells; alterations in lipid metabolism and inflammation; steatosis	[95]

Bisphenol A (BPA); Bisphenol S (BPS); Bisphenol F (BPF); Bisphenol AF (BPAF); Bisphenol B (BPB); reactive oxygen species (ROS); mitogen-activated kinases (MAPK); diabetes mellitus type 1 (DM1); diabetes mellitus type 2 (DM2); National Health and Nutritional Examination Survey (NHANES); body mass index (BMI).

## Abbreviations

BPA	Bisphenol A
BPB	Bisphenol B
BPC	Bisphenol C
BPS	Bisphenol S
BPF	Bisphenol F
BPAF	Bisphenol AF
ROS	Reactive Oxygen Species
MAPK	Mitogen-Activated Kinases
DM1	Diabetes Mellitus Type 1
DM2	Diabetes Mellitus Type 2
